# Stress-Induced Plant Specialized Metabolism: Signaling, Multi-Omics Integration, and Plant-Derived Antimicrobial Metabolites to Combat Antimicrobial Resistance

**DOI:** 10.3390/plants15020193

**Published:** 2026-01-08

**Authors:** Luis Enrique Pérez-Sánchez, Luis Mario Ayala-Guerrero, Aarón Mendieta-Moctezuma, Miguel Angel Villalobos-López, Selma Ríos-Meléndez

**Affiliations:** Centro de Investigación en Biotecnología Aplicada, Instituto Politécnico Nacional, Tepetitla de Lardizábal C.P. 90700, Tlaxcala, Mexico; lperezs2401@alumno.ipn.mx (L.E.P.-S.); layalag2200@alumno.ipn.mx (L.M.A.-G.); amendietam@ipn.mx (A.M.-M.); mvillalobosl@ipn.mx (M.A.V.-L.)

**Keywords:** specialized metabolism, physiological response (stress), biosynthetic pathways, gene regulation, multi-omics integration, RNA-seq transcriptomics, LC/GC–MS metabolomics, bryophytes, Huanglongbing (*Candidatus Liberibacter*)

## Abstract

Antimicrobial resistance (AMR) is one of the major health threats of the 21st century and demands innovative sources of bioactive compounds. In 2019, infections caused by resistant bacteria directly accounted for 1.27 million deaths and contributed to an additional 4.95 million associated deaths, underscoring the urgency of exploring new strategies. Among emerging alternatives, specialized plant metabolites stand out, as their biosynthesis is enhanced under biotic or abiotic stress. These stimuli increase reactive oxygen species (ROS), activate cascades regulated by mitogen-activated protein kinases (MAPKs), and trigger defense-related hormonal pathways involving salicylic acid (SA), jasmonic acid (JA), ethylene (ET), and abscisic acid (ABA), which in turn regulate transcription factors and biosynthetic modules, promoting the accumulation of compounds with antimicrobial activity. In this review, we synthesize recent literature (2020–2025) with emphasis on studies that report quantitative activity metrics. We integrate evidence linking stress physiology and metabolite production, summarize mechanisms of action, and propose a conceptual multi-omics pipeline, synthesized from current best practices, that combines RNA sequencing and LC/GC-MS-based metabolomics with bioinformatic tools to prioritize candidates with antimicrobial potential. We discuss elicitation strategies and green extraction, highlight bryophytes (e.g., *Pseudocrossidium replicatum*) as a differentiated chemical source, and explore citrus Huanglongbing (HLB) as a translational case study. We conclude that integrating stress physiology, multi-omics, and functional validation can accelerate the transition of stress-induced metabolites toward more sustainable and scalable medical and agricultural applications.

## 1. Introduction

Antimicrobial resistance (AMR) is a growing health threat that compromises advances in human medicine and animal health. Infections caused by bacteria, parasites and fungi have become progressively more difficult to treat because of excessive and, in many cases, inappropriate use of antimicrobials. In 2019 an estimated 1.27 million deaths were directly attributable to infections caused by resistant bacteria and 4.95 million deaths were associated with AMR [1]. If the global response is not strengthened, economic impacts by 2035 are projected to reach approximately US $412 billion per year in health-care costs and US $443 billion per year in lost productivity [2], highlighting the urgency of exploring complementary and safer strategies to conventional antibiotics.

One alternative within green biotechnology is the use of plants, sessile organisms that are continuously exposed to a wide range of biotic and abiotic stresses. In response, they have evolved complex defense mechanisms that include the synthesis of thousands of bioactive molecules derived from specialized metabolism; many of these have been reported to possess antimicrobial potential. This chemical diversity can be exploited through elicitation strategies that stimulate specific biosynthetic pathways and raise the levels of metabolites of interest, which can then be isolated as extracts or essential oils, among other formats [3].

Among the best-studied compounds are the phenolic monoterpenoids carvacrol, thymol and eugenol, as well as the phenylpropanoid cinnamaldehyde. These molecules have shown broad-spectrum antibacterial activity and, in addition, antifungal, antiviral, anti-inflammatory and even anticancer properties, mainly in preclinical models [4]. These phytochemicals act through diverse mechanisms that include membrane destabilization, enzyme inhibition, interference with biofilm formation and modulation of quorum sensing, among others [5]. Synergistic effects with conventional antibiotics have also been documented, lowering minimum inhibitory concentrations and improving therapeutic efficacy [6]. However, their safety profiles depend on dose, composition and formulation; in some cases adverse effects, including phototoxicity, have been reported, which require rigorous evaluation before any clinical or agricultural application [7,8].

**Figure 1:**
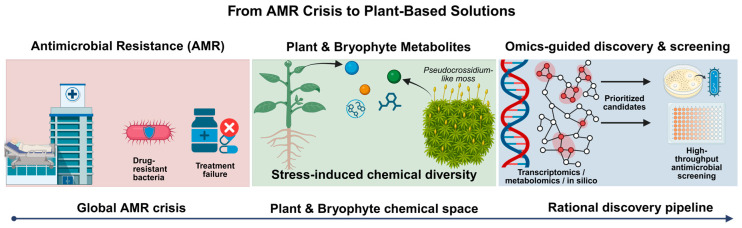
Conceptual framework linking the antimicrobial resistance (AMR) crisis to plant-based solutions. The scheme illustrates the progression from drug-resistant bacterial infections and treatment failure (**left**) to stress-induced chemical diversity in plants and bryophytes, exemplified by moss systems such as *Pseudocrossidium*. Integration of transcriptomics, metabolomics, and in silico analyses enables the prioritization of candidate compounds, which are subsequently evaluated through high-throughput antimicrobial screening (**right**).

This review compiles recent literature (2020–2025) that links plant stress with defense signaling, biosynthetic reprogramming and antimicrobial activity, prioritizing studies with comparable quantitative metrics (e.g., fold-change, minimum inhibitory concentration (MIC), fractional inhibitory concentration index (FICI) and inhibition zone diameters). We also highlight frequent inconsistencies in the literature, such as heterogeneous units (% *v*/*v* vs. µg/mL) and insufficient controls, which hinder comparison across studies. Building on this foundation, we propose a multi-omics pipeline that connects induction design (biotic/abiotic stress and elicitation), transcriptomic and metabolomic analyses, in silico prioritization of candidates and bioactivity validation. Where relevant, we emphasize evidence from underexplored lineages such as bryophytes (for example, *Pseudocrossidium replicatum*) and discuss potential agricultural applications, including the case of Huanglongbing and *Candidatus Liberibacter*. The ultimate goal is to support more rigorous scrutiny in the identification of plant metabolites with antimicrobial potential and to facilitate their transition toward medical and agricultural applications, without losing sight of safety, stability and scalability requirements (see Figure 1).

## 2. Stress-Induced Metabolic Reprogramming

### 2.1. Abiotic Stress: Quantifiable Elicitation

Abiotic stressors can positively regulate the biosynthesis of specialized metabolites and increase the abundance of molecules with antimicrobial activity. In practice, the most extensively studied abiotic stimuli include controlled water deficit, UV radiation and temperature regimes (cold or heat shock), among others [9].

Preharvest water deficit in phenolic-rich species such as olive (*Olea europaea*) has been shown to increase total polyphenol content [10]. This finding is consistent with the strong antifungal activity of these extracts against *Candida* spp., validated in broth microdilution assays [11]. Similarly, moderate postharvest exposure to UV-C in table grape (*Vitis vinifera*) induces high resveratrol accumulation in the berry skin and is associated with reduced incidence of gray mold caused by *Botrytis cinerea*; under certain regimes, the UV-C response surpasses that of UV-B, although the final effect depends on the cultivar and postharvest handling [12]. Related postharvest resistance responses have also been documented in citrus under UV-C or heat treatments [13,14].

Another example is the controlled application of temperature in *Mentha* species and its impact on the profile of foliar monoterpenes. Thermal pulses reconfigure leaf monoterpenes, and the effect on bioactivity varies according to species and conditions: in *M. arvensis*, warmer and drier conditions have increased inhibition zones against *Staphylococcus* and *Bacillus*, whereas in *M.* × *piperita* maximum effects have been observed under more temperate temperature regimes [15]. Taken together, these stimuli generate specific response chemotypes in which the basal levels of certain compounds are increased or intrinsic synergies within the extract emerge.

These stressors converge on relatively conserved signaling nodes: ROS, Ca^2+^ fluxes and MAPK cascades, which activate transcription factors (e.g., WRKY, MYB, bHLH) and thereby phenylpropanoid (PAL, C4H, 4CL), terpenoid (TPS) and polyketide (PKS) pathways [9,16,17]. The outcome is a reprogrammed chemical profile in which compounds that are absent or scarce under basal conditions emerge.

### 2.2. Biotic Stress and Elicitors: From PAMPs to Quantifiable Phytoalexins

Recognition of pathogen-associated molecular patterns (PAMPs) by pattern-recognition receptors (PRRs) triggers pattern-triggered immunity (PTI) [18]. This perception activates signaling cascades (Ca^2+^, ROS, MAPKs) that reprogram metabolism and can synergize with effector-triggered immunity (ETI) [19].

A widely studied model in *Arabidopsis thaliana* illustrates this axis: perception of bacterial PAMPs induces massive accumulation of the antifungal phytoalexin camalexin, with increases of approximately 20–60-fold over basal levels and effective control of necrotrophic pathogens in infection assays [20].

This principle translates directly to crops of agricultural interest. In soybean (*Glycine max*), elicitation or activation of key transcription factors, such as the MYB-like factor GmMYB29A2, induces the accumulation of glyceollins (I–III) and reduces disease severity caused by the oomycete *Phytophthora sojae* [21]; the magnitude of this induction has been reported to exceed 10-fold following elicitor treatment [22]. In citrus, postharvest UV-C irradiation and heat treatment induce accumulation of the phytoalexin scoparone, effectively preventing rot caused by *Penicillium* spp. [13,14]. Likewise, application of the elicitor chitosan in grapevine (*V. vinifera*) induces stilbenes such as trans-resveratrol and results in quantifiable protection against *Botrytis cinerea*, with 41–69% reductions in lesion diameter on leaves [23].

Taken together, these examples support the notion that exposure of plants to biotic or abiotic external signals, as well as to exogenous elicitors, provokes chemical changes that are detectable and measurable using comparable quantitative metrics (e.g., metabolite fold-change, minimum inhibitory concentration (MIC), IC_50_, EC_50_ and percentage reduction in disease severity), which translate into effective responses against pathogens.

### 2.3. Hormonal Regulation, Transcription Factors and Crosstalk: The Conductors of the Defensive Orchestra

Early signals are amplified by second messengers such as Ca^2+^, ROS and MAPKs, which function as an “alarm” and report exposure to stress; downstream, regulation is mediated by phytohormones that orchestrate the specific pathway leading to the metabolic response. The SA/NPR1 pathway is mainly associated with biotrophs and immune memory. SA activates the receptor NON-EXPRESSOR OF PR-1 (NPR1), which undergoes redox-dependent changes and migrates to the nucleus to cooperate with transcription factors (TFs) of the TGA and WRKY families, establishing an effective defense against biotrophic pathogens. This pathway is central to Systemic Acquired Resistance (SAR), a form of “memory” that primes distal tissues for future challenges [24,25].

In addition, necrotrophic pathogens and herbivores stimulate the JA/ET pathway, in which the bioactive signal jasmonoyl-isoleucine (JA-Ile) promotes COI1–JAZ interaction and JAZ degradation, releasing MYC2 and other bHLH factors to induce protective responses (e.g., terpenoids, alkaloids) [26]. In parallel, JA and ET converge on ERF-like factors; in Arabidopsis, *ORA59* integrates this synergy and regulates genes such as *PDF1.2*, which plays a key role against necrotrophic pathogens [27]. Antagonism between the SA and JA/ET pathways has been reported, although the outcome depends on context (tissue, dose, time-of-day biology and pathogen). The underlying interaction network reveals multiple points of crosstalk (protein stability, transcriptional control, hormonal homeostasis) that can explain scenarios of antagonism, variable activity or synergy [28].

**Figure 2:**
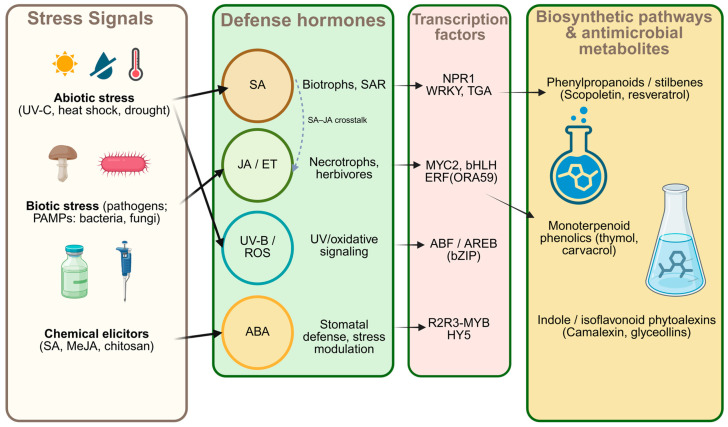
Schematic representation of stress perception, hormone signaling, transcriptional regulation, and biosynthetic pathways involved in the production of antimicrobial plant metabolites. Hormones and transcription factors shown represent major defense modules activated under abiotic and biotic stress. Arrow notation: solid arrows indicate directional signaling/putative positive regulatory links; the dotted arrow indicates SA–JA/ET pathway crosstalk.

In the case of ABA, its role has traditionally been associated with drought, and it modulates stomatal defense: it can promote rapid closure that limits bacterial entry, while also reconfiguring responses according to water status and other signals, acting either as an attenuator or a potentiator [29]. The hormonal pathways mentioned above act as “conductors of the orchestra” that instruct specific groups of “managers” what to do. These managers are TFs—proteins that function as master switches to “turn on” or “turn off” metabolite factories (biosynthetic pathways). Each hormonal pathway has its own characteristic TFs: the SA pathway activates WRKY and TGA families; the JA pathway activates MYC2; JA/ET synergy activates ERFs; ABA signals through ABF/AREB; and UV stress pathways activate the MYB family (see Figure 2). Table 1 summarizes metabolites with antimicrobial activity and their predominant hormonal regulators.

It is crucial to note that the magnitude and type of metabolic response are not universal; the final chemical profile is highly dependent on key modulatory factors, including genotype, specific tissue, developmental stage (time) and environment [34,35]. This contrast is particularly evident between controlled laboratory conditions, and the multiple, unpredictable stressors present in the field [36]. Taken together, these variables, along with heterogeneity in extraction and assay methods, help explain the wide variability in reported antimicrobial potency (e.g., inconsistent MIC values) across the literature [37].

## 3. Classes of Metabolites and Mechanisms of Action

Having established how plants activate their chemical arsenal in response to stress, this section focuses on which specific “weapons” make up that arsenal.

The chemical biodiversity of plants offers an immense set of metabolomic responses tailored to different stimuli, yet most reported metabolites with antimicrobial activity cluster into a few major chemical “superfamilies”, such as terpenoids, phenylpropanoids (including flavonoids and stilbenes), alkaloids and saponins.

Each family tends to display characteristic mechanisms of action. For example, the phenolic monoterpenes carvacrol and thymol are known to destabilize bacterial membranes and alter ionic permeability, whereas alkaloids such as berberine can intercalate into DNA or inhibit efflux pumps (EPIs), thereby enhancing the activity of other antibiotics. Table 2 summarizes these main chemical families, their emblematic compounds, their best-known mechanisms of action, examples of microbial targets and the reported activity ranges (MIC/MBIC/ED_50_).

## 4. Multi-Omics and Bioinformatics for Discovery and Prioritization

### 4.1. Pipeline: From RNA-Seq + LC/GC-MS to DEG–Metabolite Correlations

**Figure 3:**
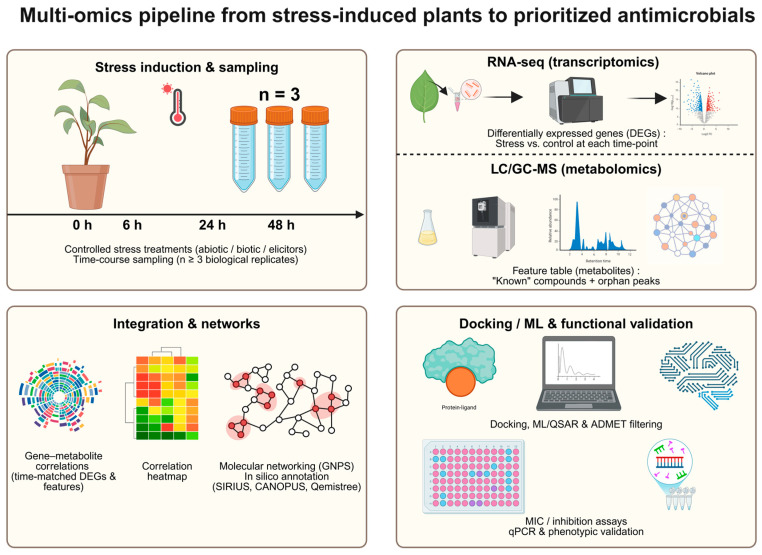
Workflow illustrating the integration of transcriptomics, metabolomics, and in silico analyses to prioritize antimicrobial metabolites from stress-induced plant systems.

Here we outline a conceptual workflow, synthesized from current best practices, to guide the integration of transcriptomics (RNA-seq) and LC/GC-MS-based metabolomics for prioritizing candidates with antimicrobial potential for downstream validation. A robust current approach integrates multi-omics datasets to predict biosynthetic pathways de novo [57]. This pipeline focuses on genes associated with a given biosynthetic route and complements their analysis with transcriptomics (RNA-seq) and metabolomics (LC-MS or GC-MS) to identify bioactive compounds of interest [58]. To robustly link gene induction to the accumulation of metabolites with antimicrobial potential, an appropriate experimental design is essential. This implies serial sampling at selected time points (e.g., 0, 6, 24, 48 h) after application of the elicitor or stimulus of interest (biotic or abiotic stress), with a minimum of three biological replicates (*n* ≥ 3) per independent experiment [59]. Samples for transcriptomic and metabolomic analyses must be collected from the same tissue type, under the same conditions (photoperiod, developmental stage, etc.) and at the same time points to ensure that the datasets are directly comparable and suitable for correlation analyses (see Figure 3).

Each arm of the pipeline generates a different type of data. The transcriptomic arm (RNA-seq) yields list of differentially expressed genes (DEGs), identifying which genes are “turned on” or “off” together with their magnitude (log_2_FC) and statistical significance (FDR) at each time point [59]. In parallel, the metabolomic arm detects and annotates the peaks of metabolites that have accumulated or decreased, many of which may initially be unknown (“orphan peaks”) [60]. After preprocessing, this results in paired quantitative matrices of genes and metabolites across time and conditions.

The strength of the pipeline lies in the bioinformatic correlation of these two matrices [57,58]. The objective is to identify patterns of co-expression and co-accumulation: a gene that encodes a biosynthetic enzyme—such as a cytochrome P450 (CYP450) or a UDP-glycosyltransferase (UGT) that switches “on” (high fold-change) exactly when a specialized metabolite for example, a new terpene “accumulates” (high intensity). This temporal coincidence constitutes one of the strongest lines of evidence to propose that the gene encodes the enzyme responsible for the formation of the corresponding metabolite. Prioritized candidates can then advance to annotation (e.g., MSI Level 2) and experimental validation (qPCR, chemical standards, antimicrobial activity assays) [60].

### 4.2. Analytics and Networks: Annotation, Metabolomic Networking and Co-Expression

The pipeline described in Section 4.1 generates two matrices, transcript counts and chromatographic features and this is where the main challenge begins. We now have signal detection, but we still need to “assign surnames” through chemical annotation or identification, especially for the “orphan peaks” that lack matches in spectral libraries [61].

However, it is important to recognize that current MS/MS spectral libraries remain incomplete and unevenly populated, with biases toward well-studied organisms and compound classes, as well as method-dependent effects (instrument type, collision energy, ionization/adduct patterns) that can limit the transferability of spectral matches across studies. Consequently, many plant specialized metabolites particularly low-abundance features, conjugates, and closely related isomers remain unmatched, and even apparent library hits may require cautious interpretation. In this context, combining library searching with network context and state-of-the-art in silico annotation helps reduce over-interpretation while prioritizing candidates for targeted confirmation. Importantly, broader community sharing of MS/MS data and associated metadata through open platforms accelerates library growth and curation, improving reusability and progressively converting ‘orphan peaks’ into identifiable metabolites over time [61,62,63].

For this reason, the field has moved from classical feature processing (e.g., MZmine [64], MS-DIAL [50]) toward network-based analytics that organize the apparent chaos and add biological context.

The most transformative approach has been Molecular Networking, implemented in the GNPS environment [62]. This method connects spectra based on similarity, so that “chemical families” emerge, where a known compound “pulls the thread” of related unknowns. In other words, we move from seeing isolated stars to recognizing constellations, grouping known and unknown metabolites based on MS/MS fragmentation similarity. In parallel, tools such as Qemistree [65] project chemical space as a tree of molecular “fingerprints” that can be crossed with metadata (tissue, time) and expression data (co-expression), linking chemistry and biology on the same map.

To name unknown nodes in these networks, state-of-the-art in silico tools are employed. Platforms such as SIRIUS/CANOPUS [51] and COSMIC [63] use deep learning to predict the formula and structural class of a metabolite from its spectrum. In parallel, spectral similarity algorithms such as Spec2Vec [66] and MS2Query [67] dramatically improve the ability to find structural analogs that are not present in libraries.

These co-dynamics of co-expression and co-accumulation make it possible to identify candidate enzymes and propose pathways, generating solid hypotheses before targeted validation (chemical standards, qPCR, bioassays) [57,58,60]. In other words, it is akin to assigning a “provisional ID” to peaks: a tentative name, a structural class and the chemical and genetic “neighborhood” that supports it. These open-source tools allow researchers to navigate the metabolomic “dark matter” and prioritize which orphan peaks are biosynthetically relevant [68] (see Table 3).

### 4.3. Functional Prediction (Docking/ML) and Prioritization Criteria

Once the annotation stage has provided candidates (the “provisional IDs”), the key question is no longer what is there? but what is most reliable and worth testing first? This is where functional prediction becomes central for prioritization. The aim is to filter, in silico, hundreds of “orphan” metabolites and select the few that truly justify costly validation in the laboratory. The main tool for this is molecular docking, which acts as a prioritization funnel by simulating whether the “key” (metabolite) fits into the “lock” (protein target) and by predicting its relative binding affinity [69].

In parallel, machine learning (ML) and QSAR models employ artificial intelligence to recognize patterns between chemical structure and biological activity [55], thus accelerating candidate identification. Crucially, they are also used to estimate ADMET profiles (Absorption, Distribution, Metabolism, Excretion, Toxicity), which are fundamental for prioritization and do not replace bioassays [70].

However, it is essential to adopt the critical perspective emphasized in this review: in silico results are not proof of activity, but tools for prioritization. For natural products, in silico methods are most powerful when they shrink the search space (combining docking, similarity and ADMET rules), yet they can easily overpromise if uncertainty is not reported (for example, variance among scores). Docking scores do not always correlate directly with MIC values obtained in the laboratory. There are significant challenges (protein flexibility, solvation, target selection) that can lead to false positives [56]. Therefore, to make these methods genuinely useful, they must follow rigorous protocols, which are summarized in Box 1.

Box 1 **Best practices for in silico screening of natural products.**

1. Receptor (target) preparation

- Use high-resolution crystallographic or cryo-EM structures (≤2.5 Å) whenever available.
- For AlphaFold models, evaluate quality using pLDDT, Ramachandran plots, and visual inspection before use.
- Adjust protonation states of catalytic residues and retain only functional cofactors.
- When possible, consider multiple receptor conformations (ensemble docking) to capture flexibility [71,72].

2. Ligand preparation (natural metabolites)

- Generate relevant tautomers and protonation states at physiological pH (7.0–7.4).
- Minimize energy and sample realistic conformations, especially for highly flexible molecules (e.g., terpenes).
- Define stereochemistry and 3D geometry correctly before docking and check for artifacts in very lipophilic compounds [73,74].

3. Binding site definition

- Prioritize docking directed to a validated active site (crystallographic data, mutagenesis, or other functional evidence).
- Define the grid box with a margin of ~5 Å around key residues.
- Avoid relying exclusively on blind docking due to its low specificity, and consider structural waters when they participate in catalysis [75,76].

4. Docking protocol validation

- Perform redocking of the co-crystallized ligand and require a root-mean-square deviation (RMSD) ≤ 2.0 Å to validate the methodology.
- Use positive controls (known inhibitors) and negative controls (decoys), and evaluate the protocol’s ability to separate actives from inactives (AUC, enrichment factor, EF).
- Report full parameters: software and version, grid size, exhaustiveness, number of runs, etc. [77,78,79].

5. Critical analysis of results

- Do not select candidates solely by score; examine interactions, geometry, and chemical plausibility.
- Visualize hydrogen bonds, hydrophobic contacts, π–π interactions, and salt bridges, and analyze pose clusters.
- When resources allow, refine the top candidates using short molecular dynamics simulations (5–20 ns) or rescoring with MM-GBSA/MM-PBSA [80,81,82].

6. Final prioritization of candidates

- Combine calculated affinity with pose stability and the presence of specific interactions relevant to the target.
- Filter according to ADMET properties and discard clearly reactive or problematic compounds.
- Avoid candidates with extreme lipophilicity (e.g., LogP > 6) or unstable geometry, and advance only those that clearly justify experimental validation (MIC, enzymatic assays, etc.) [83,84,85].
  Finally, docking should be interpreted as a hypothesis generating prioritization step rather than a standalone proof of bioactivity. Top-ranked compounds should then be advanced to orthogonal experimental validation when feasible, through target-level biochemical/biophysical assays, and in all cases through antimicrobial phenotypic testing (e.g., MIC, IC50 or growth/viability readouts) with appropriate positive controls and concentration ranges compatible with solubility. This closes the loop from in silico ranking to actionable, experimentally supported candidates.

However, several limitations should be acknowledged, along with strategies to balance comprehensiveness and objectivity. Despite their power, multi-omics pipelines are susceptible to biases that can inflate confidence if not explicitly controlled. At the data level, batch effects, uneven sampling depth, missing values, and heterogeneous acquisition settings can distort integrative analyses, while annotation uncertainty may propagate through networks and correlation-based links. At the interpretation level, co-expression and co-accumulation relationships are not inherently causal, and integrative models can overfit when the same datasets are used for both model selection and performance assessment without independent validation [57,58,60]. In silico prioritization also carries a non-trivial false-positive risk: docking scores are sensitive to target selection, protein flexibility, solvation, and scoring-function limitations, and therefore may not correlate directly with experimental potency [56]. 

To mitigate these issues, we recommend:

1. rigorous quality control and batch-aware processing.
2. transparent reporting of annotation confidence and model uncertainty.
3. conservative, pre-defined filtering criteria, and sensitivity analyses.
4. orthogonal validation with appropriate controls (bioassays, chemical standards, or target-level assays when feasible) before making strong functional claims [56,57,58,60].

Together, these practices improve robustness and reproducibility.

## 5. From Induction to Application: Experimental and Translational Frameworks

### 5.1. Elicitation and Culture Systems for Metabolite Production

Having established throughout this review that stressors enhance the biosynthesis of specialized metabolites [8], the next step is to address how to quantify them and how to achieve controlled, scalable production of metabolites of interest [86]. A nice peak in the chromatogram is not a result, but a promise; for a metabolite to advance as an antimicrobial candidate it must become tangible, reproducible and available in sufficient quantities to be evaluated rigorously. In plant cell and tissue cultures, elicitors are the signals that “trick” cultures into activating their defense pathways [87,88].

This approach is particularly powerful with key hormones: methyl jasmonate (MeJA) and salicylic acid (SA) are among the most reliable elicitors to switch on metabolite pathways in cell and tissue cultures and boost metabolite production [89]. Likewise, biotic elicitors such as chitosan (derived from fungal chitin) have been established as potent inducers [90]. Recent reviews already provide both useful dose ranges and common pitfalls (e.g., non-linear dose–response curves, narrow temporal windows) [87,89,90]. In general, MeJA/SA tend to trigger phenolic and terpenoid pathways with good outcomes, whereas chitosan offers an economical, biocompatible route that can also improve tolerance and performance of the system being scaled up [89,90].

The true impact of elicitation is realized when it is combined with biotechnological culture platforms. Instead of relying on field harvests (highly variable), production is shifted to in vitro culture systems such as cell suspension cultures, organ cultures (e.g., hairy roots or adventitious roots) and bioreactors [91,92]. These systems offer scalability and tight control. Bioreactors (cultivation tanks), for example, are being optimized for large-scale root cultures, enabling continuous industrial production of specialized metabolites independent of climatic conditions [93,94]. Hairy roots, generated via transformation with *Rhizobium rhizogenes*, combine biosynthetic stability with compatibility with various bioreactor formats (temporary immersion, mist systems, etc.), and have shown competitive yields for complex phytocompounds [92,94].

Thus, this section functions as the first decisive filter: here we define what to elicit, in which culture system and under which parameters. These decisions determine the amount, reproducibility and comparability of the biomass, and everything that follows formulation, stability and antimicrobial validation will depend on three foundations being consolidated at this initial stage: sufficient production, clear traceability and consistency between batches [88].

### 5.2. Extraction, Formulation, Stability and Assay Panels Against Phytopathogens

The previous section showed how to obtain cellular biomass, but this raw material now needs to be processed so that it becomes useful, stable and comparable. As we already know, a nice peak in the chromatogram is not a result but a promise; for a metabolite to progress as an antimicrobial candidate, we must first make sure we extract it properly, “dress” it so that it survives, and demonstrate that it works. In plant cell and tissue systems, this means bringing the molecule from the culture flask to formats that can be tested reproducibly in bioassays.

The first challenge we face is extraction. We must first decide what we want (a fraction/a target compound) and then how to obtain it: if the candidate is volatile, we avoid heat; if it is phenolic, we protect pH and compatibility with LC/GC. Moving away from toxic organic solvents, the modern approach is “green extraction” [95], which uses technologies such as microwave-assisted extraction (MAE) [96], ultrasound-assisted extraction (UAE) [97] or supercritical CO_2_ extraction [98]. These methods stand out for their yields and reduced environmental footprint; they already have well-standardized protocols and, importantly, open the door to developing new biocompatible solvents, such as natural deep eutectic solvents (NADES), which are highly effective for selectively extracting phytochemicals [99,100,101].

The next challenge is formulation: how to deliver these compounds to where they can actually act. Most antimicrobial metabolites (such as those in Table 2) are hydrophobic and unstable—that is, they do not mix well with water and degrade rapidly—making direct use difficult. For this reason, a major current focus is on finding delivery systems and encapsulation strategies—vehicles capable of encapsulating and protecting them [102,103]. The most promising strategies include nanoemulsions (often produced by ultrasound) [104,105,106], liposomes [107] and cyclodextrin inclusion complexes [108]; these systems improve solubility, stabilize the compound and enhance its antimicrobial activity. Nevertheless, in laboratories with tight budgets or in early exploratory assays, surfactants such as Tween 80 offer a practical alternative: they solubilize the hydrophobic compound, provide minimal stabilization and allow preliminary testing without advanced systems [109].

Once the metabolite has been produced, extracted and formulated, the remaining step is validation. This is the point at which methods become less uniform and differences between studies become more evident. Therefore, to ensure that results are comparable, it is essential to adhere to a standardized assay panel:

1. How to measure it (comparability)
  - Core metric: MIC by broth microdilution.
  - Standards: CLSI (M07, M27, M38) and EUCAST [110,111,112].
  - Combinations: FICI via checkerboard assays [113].
2. Frequent pitfalls (main sources of noise)
  - Inconsistent units: µg/mL vs. % *v*/*v*.
  - Irregular reporting of MIC_90_.
  - Variation in vehicles: DMSO, ethanol and Tween 80 must be fixed and reported.
  - Require n ≥ 3 biological replicates.
3. Advanced metrics
  - Biofilms: MBIC/MBEC [114].
  - Quorum sensing: reporter strains (e.g., *Chromobacterium violaceum*) [115].
  - Phytopathogens: progress from plate assays to detached-leaf tests or whole-plant systems [116].

### 5.3. From Lab to Field: Translational Challenges and the Bryophyte Frontier

We now move to the real challenge: going from the flask to the field. As discussed in previous sections, a nice peak is not a product. We know we can produce and formulate metabolites in the laboratory, but the translational gap is the “final boss” where most candidates fail. To move forward, regulatory agencies demand three clear elements: chemical identity and batch-to-batch consistency, safety (for humans and the environment), and efficacy under realistic conditions.

For agricultural use as a “biopesticide”, one must navigate the rigorous frameworks of agencies such as the EPA in the United States (which regulates biochemical pesticides) and the European procedure for “basic substances” under Regulation (EC) No 1107/2009, alongside OECD guidance for botanical active substances used in plant protection products [117,118,119]. Beyond regulatory compliance, translational deployment must also address formulation stability, batch-to-batch variability, and efficacy under realistic conditions constraints widely discussed for plant-derived biopesticides such as essential oils [120].

For pharmaceutical use, botanical products must comply with drug-development regulations. The FDA guidance on botanical drug development emphasizes rigorous chemistry, manufacturing and controls (CMC), batch-to-batch consistency, GMP, and staged nonclinical/clinical evaluation via IND/NDA pathways requirements that are distinct from agricultural registration and should therefore be considered independently [121].

The next challenge is to find genuinely novel chemical sources. Much of the research has traditionally focused on angiosperms (flowering plants). This is where bryophytes (mosses and liverworts) emerge as an underexplored frontier [122]. Their value is not only historical or taxonomic; it is chemical and functional. Mosses and liverworts bring together two qualities that are hard to find in combination: unusual chemistry and highly inducible defense responses. Despite their small size, bryophytes possess a unique and powerful chemical repertoire, rich in terpenoids, bisbibenzyls and phenolic compounds, with antimicrobial activity against *Candida*, *Staphylococcus* and even oomycetes [123,124]. Recent syntheses focusing on bryophyte essential oils and volatile compounds further underscore this underexplored chemical space [125]. Clearly, much remains to be discovered in these lineages, which makes them particularly attractive; many of their biosynthetic pathways remain mysterious at the gene level, opening the door to multi-omics discovery strategies [119,126].

Crucially, bryophytes also respond to stress (abiotic and biotic) by reprogramming their metabolism [127]. In fact, evolutionarily ancient lineages such as mosses and liverworts have developed unique pigment/flavonoid classes such as auronidins, which do not occur in flowering plants and are implicated in defense, cell wall modification and stress tolerance [128,129]. The appearance of auronidins is often a sign that the defense system has been activated; this is important because it provides a useful signal to schedule sampling (0–6–24–48 h) and capture precisely the time windows in which genes and metabolites begin to respond.

This metabolic plasticity, combined with their suitability for in vitro culture (as in our model *P. replicatum*), which exhibits full desiccation tolerance and strong responses to inducers [130], allows us to control when to trigger metabolic peaks. Moreover, recent studies in bryophyte models (*Marchantia*, *Physcomitrium*) reveal lipid and phenolic profiles rich enough to fully justify the growing interest in these systems: there is substantial chemical diversity to explore, and current tools (GNPS, SIRIUS, Spec2Vec and modern analytical workflows) will allow this space to be mapped with precision [51,66,131,132].

### 5.4. HLB as a Proof of Concept: When the Pathogen Does Not Grow on Plates

Finally, to illustrate the power of the multi-omics pipeline described in this review, we apply it to one of the most urgent and difficult phytosanitary challenges (a true wicked problem): Huanglongbing (HLB), or citrus greening disease. This disease, primarily associated with the fastidious bacterium *Candidatus Liberibacter asiaticus* (CLas) [71], has become practically pandemic, causing losses in yield, fruit quality and orchard viability that threaten the sustainability of the citrus industry in key regions of the Americas, Asia and the Mediterranean [71]. In this scenario, the debate is no longer whether the economic impact is important, but rather how long citrus cultivation will remain profitable without innovative management strategies.

The challenge of controlling HLB is twofold. First, CLas is a Gram-negative α-proteobacterium that is unculturable in standard laboratory media and strictly confined to the phloem, where it is protected from topical treatments and transmitted by the Asian citrus psyllid *Diaphorina citri* [72]. Second, the inability to culture CLas prevents the application of a classical antibiotic discovery scheme: there are no Petri dishes with CLas, no growth curves on which to test compound libraries. This makes the “assay panel” described in Section 5.2 insufficient on its own for the discovery of new drugs.

**Figure 4:**
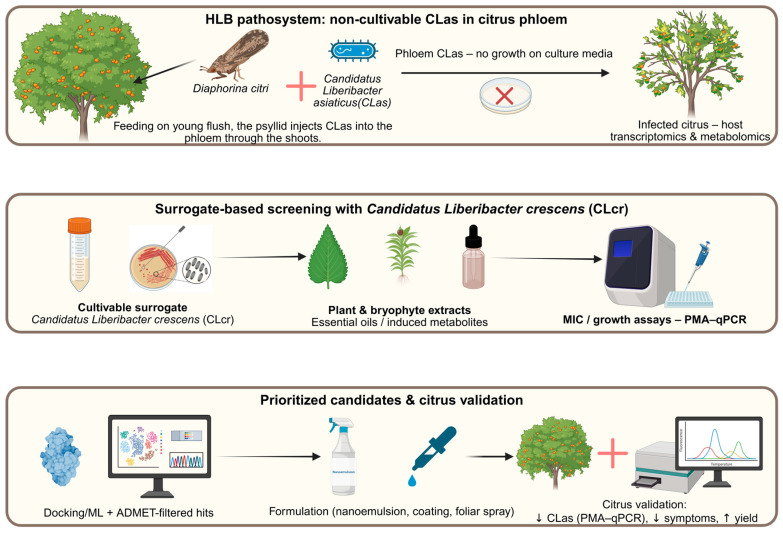
Conceptual multi-omics pipeline for Huanglongbing (HLB): top panel, non-cultivable *Candidatus Liberibacter asiaticus* (CLas) in citrus phloem, transmitted by *Diaphorina citri*; middle panel, surrogate screening with cultivable *Candidatus Liberibacter crescens* (CLcr) and plant/bryophyte extracts to identify inhibitory candidates; bottom panel, prioritized hits formulated (nanoemulsions, coatings, sprays) and validated in infected citrus (PMA-qPCR, symptoms, yield).

This is precisely where the multi-omics pipeline becomes essential (Figure 4).

To overcome the obstacle of an unculturable pathogen, there are two complementary strategies: (i) reconstruct its biology from host omics (transcriptomics and metabolomics of infected citrus) and (ii) develop cultivable surrogate models that allow screening assays and mechanistic studies. In this context, research has focused on *Candidatus* L. *crescens* BT-1 (CLcr), the first cultivable member of the *Liberibacter* genus [73].

This microorganism shares a substantial core of essential functions and genome organization with pathogenic *Liberibacter* species and has been proposed as a surrogate model for basic biology, functional genomics and antimicrobial screening [73]. Inhibition assays, mutagenesis and metabolic network analyses in *L. crescens* make it possible to explore targets and pathways whose direct manipulation in CLas would be unfeasible, but which can be extrapolated by orthology and pathway conservation. Recent work with natural products exemplifies how this strategy is implemented.

On one hand, plant extracts and metabolites produced by citrus endophytes have been evaluated using systems that combine in vitro assays with viability analysis by PMA-qPCR on leaf discs from infected trees. These studies show that extracts from aromatic plants (e.g., oregano, cinnamon, turmeric, thyme) and metabolites produced by endophytes such as *Bacillus amyloliquefaciens* significantly reduce CLas viability in plant, following a workflow that closely mirrors our pipeline: initial screening in cultivable models, followed by validation in infected citrus tissues [72].

On the other hand, recent studies have gone a step further: they have used spatial metabolomics to map chemicals in citrus tissues, employed *L. crescens* as a proxy for in vitro screening, and validated the hits in infected roots. Based on these spatial maps, metabolic modeling and in vitro assays, it was demonstrated that ferulic acid and several citrus flavonoids are highly bactericidal against CLas, with potency comparable to oxytetracycline, and that they also inhibit *L. crescens* growth in diffusion assays [74]. In essence, the process begins with the diseased tree, identifies candidate metabolites via omics and then validates their activity using a cultivable model organism together with assays in infected systems, following the same pipeline proposed in this review.

This case study with “difficult” pathogens shows that the overall strategy must change. We need to integrate elicitation, explore new chemical sources (such as bryophytes) and use multi-omics pipelines to identify and validate compounds in silico and in vitro, adopting “green” and sustainable development strategies [75]. In this context, bryophytes fit naturally. The same scheme applied to the citrus microbiome and the metabolome of infected trees can be extended to mosses such as *P. replicatum*: inducing them with defense phytohormones (SA, ABA, JA), capturing the transcriptomic–metabolomic window, prioritizing biosynthetic pathways associated with antimicrobial metabolites and, finally, testing those extracts or enriched fractions against *L. crescens*.

Integrating these results with docking and ML/QSAR models against essential CLas targets would yield a “shortlist” of candidate molecules filtered by theoretical affinity, ADMET properties and sustainability criteria (Box 1). These molecules could in turn be validated in infected citrus systems, replicating the logic already demonstrated for ferulic acid and flavonoids [73,74]. In this way, HLB serves not only as a citrus case where new alternatives are urgently needed, but also as proof that multi-omics combinations, stress priming and formulation of natural products can offer realistic routes to more sustainable therapies.

For underexplored chemical sources such as bryophytes, these systems provide fertile ground where the goal is not to compete with already described citrus compounds, but to add information to the repertoire of antimicrobial and defense-modulating metabolites that, together, may enable the design of integrated strategies against unculturable pathogens such as CLas.

## 6. Conclusions and Perspectives

This review has traced a path from the global crisis of antimicrobial resistance (AMR) to plants and, in particular, bryophytes as reservoirs of defensive metabolites. Recent evidence shows that many of these compounds are not static, but the result of stress-triggered metabolic reprogramming finely regulated by hormonal networks (SA, JA/ET, ABA) and transcription factors that respond to biotic and abiotic cues. Understanding this logic allows us to move beyond “trial-and-error” elicitation and toward more rational treatments aimed at activating specific pathways and chemotypes of interest.

Chemically, much of the actual diversity is concentrated in a few major superfamilies—terpenoids, phenylpropanoids, alkaloids and saponins—but their mechanisms of action are varied and often complementary: membrane permeabilization, cell wall disorganization, interference with biofilms and quorum sensing, inhibition of enzymatic targets or efflux pumps, among others. The literature suggests a complex reality in which the apparent antimicrobial potency depends not only on the metabolite type, but also on context: genotype, tissue, developmental stage, stress regime, extraction method and formulation. On top of this, the lack of standardization in units, controls and metrics (MIC, MBIC, FICI, % reduction in disease severity) remains a major obstacle for comparing studies and objectively prioritizing candidates.

The multi-omics component offers a way forward. Integrating RNA-seq with LC/GC-MS, molecular networking and in silico tools (metabolite annotation, docking, ML/QSAR models) can help connect biosynthetic genes with specialized metabolites and measurable antimicrobial phenotypes, instead of merely accumulating long lists of genes and chromatographic peaks. The key challenge is to filter noise to uncover causal relationships. The pipeline proposed here focuses on identifying functional gene–metabolite pairs, supported by co-expression and co-accumulation. This approach helps to discard experimental noise, narrow down the number of candidates and facilitate the transition into formulation stages (nanoemulsions, liposomes, green extraction) and testing in complex systems.

Bryophytes emerge in this context as a relatively unexplored but particularly promising chemical space. Their extreme desiccation tolerance and the ease with which their responses can be induced by phytohormones or controlled stresses make them ideal models to study how stress priming reshapes the metabolome. Species such as *P. replicatum* illustrate how combinations of treatments (SA, JA, ABA), multi-omics and bioassays can reveal metabolites with activity comparable or complementary to reference essential oils, with the added advantage of providing novel structures that are less exploited by industry.

The case of Huanglongbing (HLB) encapsulates the relevance of this approach for difficult phytosanitary problems, where the main pathogen is unculturable and current control schemes are unsustainable. In this scenario, the combination of metabolomics in citrus, the use of *L. crescens* as a surrogate model, and the validation of natural compounds has shown that it is possible to identify molecules with potency comparable to currently used antibiotics. Extending this logic to metabolites derived from bryophytes or other plant sources integrating stress design, omics, in vitro screening and testing in infected systems opens a realistic path toward greener and more resilient management strategies.

Looking ahead, the challenge is no longer to prove that plants and bryophytes can produce potent antimicrobial metabolites, but to build reproducible pipelines that integrate rational elicitation design, multi-omics and network analysis, quantitative and standardized bioassays, formulations that optimize stability, and evaluation in agricultural and clinical scenarios within a One Health framework. Progress along these lines will help ensure that the molecules reviewed here cease to be merely laboratory examples and become components of integrated strategies to confront resistant and unculturable pathogens, contributing to a transition toward more sustainable antimicrobials with a lower ecological footprint.

## Figures and Tables

**Table 1:** Hormones, transcription factors (TFs), nodes and metabolic pathways associated with plant defense responses.

Inducing Hormone (Signaling Pathway)	TF Family	Receptor/Key node	Target Genes	Metabolites	Context	Reference
SA	WRKY/TGA	NPR1	*PR1*, *ICS1* (*SID2*), *PAL*; PAL/CHS	Phytoalexins and phenylpropanoids; SAR	Biotrophic pathogens	[24,25,30,31]
JA (JA-Ile)	MYC2 (bHLH)	COI1–JAZ (MYC2 derepression)	*VSP2*, *LOX2*, *TPS*; *DXS*, *HMGR*	Terpenoids and alkaloids; ISR	Necrotrophic pathogens/herbivores	[32]
JA–ET signaling synergy	ERF (p. ej., *ORA59*)	COI1–JAZ + EIN2/EIN3	*PDF1.2*, *PR4*, *CHI*	JA/ET-dependent antimicrobial programs	Necrotrophs; wounding	[33]
ABA	ABF/AREB (bZIP)	PYR/PYL–PP2C–SnRK2	*RD29B*, *RAB18* (modulate SA/JA/ET)	Stomatal immunity, osmotic adjustment; modulatory role	Water deficit/combined stress	[29]

Notes: TF, transcription factor; PAL/CHS, entry-point genes of the phenylpropanoid/flavonoid pathway; LOX2/TPS, oxylipin/terpenoid pathway genes; PDF1.2, antimicrobial defense response genes; ISR/SAR, induced systemic resistance (JA/ET) and systemic acquired resistance (SA), respectively.

**Table 2:** Chemical families, emblematic compounds, mechanisms of action, examples of microbial targets and reported activity ranges (MIC/MBIC/ED_50_).

Chemical Family	Representative Compounds	Mechanism of Action	Spectrum/ Targets	Typical Metric	References
Phenolic monoterpenoids (terpenoids)	Carvacrol, thymol	Membrane permeabilization; collapse of ΔΨ/ΔpH; anti-quorum sensing activity; biofilm reduction	*E. coli*, *S. aureus*, *Candida albicans*	MIC 125–1000 µg/mL (broth microdilution)	[4,5,38]
Phenylpropanoids	Cinnamaldehyde, eugenol	Protein–membrane interactions; inhibition of FtsZ/ATPase; oxidative stress induction; anti-biofilm activity	*Listeria monocytogenes*, *B. cinerea*, *Vibrio* spp.	MIC 250–1000 µg/mL	[6,39,40]
Flavonoids	Quercetin, kaempferol	Inhibition of DNA gyrase/topoisomerases; membrane modulation; anti-quorum sensing and anti-biofilm activity	*P. aeruginosa*, *S. aureus*	MIC 50–500 µg/mL	[38,41,42,43]
Alkaloids	Berberine, sanguinarine	DNA intercalation; inhibition of FtsZ/topoisomerases; substrates of efflux pumps (enhanced synergy with EPIs)	*Bacillus subtilis*, *S. aureus* (MRSA)	MIC 16–256 µg/mL; synergy with EPIs	[39,44,45]
Saponins (triterpenoid)	β-Aescin	Complexation with ergosterol leading to pore formation; membrane permeabilization	*C. albicans*, *Fusarium* spp.	MIC 50–400 µg/mL	[46,47,48]
Stilbenes (phytoalexins)	Resveratrol	Anti-quorum sensing and anti-biofilm activity; ROS induction; membrane permeabilization; antifungal effects	Bacteria	MIC 50–200 µg/mL	[12,49,50]
Coumarins (phytoalexins)	Scopoletin/scoparone	Inhibition of growth and spore germination; interference with respiration and cell wall integrity	*Phytophthora* spp.	ED_50_ (germination/growth)	[13,14,51]
Isothiocyanates (from glucosinolates)	Allyl ITC, benzyl ITC	Electrophiles reacting with thiol groups; enzyme inactivation; oxidative stress induction	Gram-positive and Gram-negative bacteria/fungi	MIC 50–300 µg/mL	[52,53,54]
Legume phytoalexins	Glyceollins I–III	Antifungal activity; multi-target effects (membrane and enzymatic disruption); anti-biofilm activity	*Fusarium*, *Botrytis*, *Phytophthora*	MIC 25–750 µg/mL; 10.9–61% inhibition	[21,22,55]
Indole alkylamines (Brassicaceae)	Camalexin	Antifungal activity: accumulation increases 5–10× upon flg22 elicitation	*Botrytis*, *Alternaria*	Lesion reduction; strain-specific MICs reported	[20,30,56]

Note: MIC, minimum inhibitory concentration; MBIC, minimum biofilm inhibitory concentration; ED_50_, median effective dose. MIC and MBIC values are reported in µg/mL unless otherwise indicated. ED_50_ refers to the concentration required to reduce growth, germination, or disease-related parameters by 50%, depending on the assay. ΔΨ, membrane potential; ΔpH, transmembrane proton gradient; EPIs, efflux pump inhibitors. Activity ranges are indicative and may vary depending on the microorganism, strain, assay conditions, and experimental setup.

**Table 3:** Representative bioinformatics tools across the multi-omics workflow and their primary applications.

Workflow Step	Tools	Primary Application	Typical Output
Feature detection & alignment	MZmine (v3.6) [64]; MS-DIAL (v4.90) [50]	Peak picking, deconvolution, alignment, feature table generation	Feature table (*m*/*z*, RT, intensity)
MS/MS similarity networking	GNPS [62]	Spectral networks, chemical families, annotation propagation	Molecular network graph
Network visualization/curation	Cytoscape (v3.10)	Visualization, filtering, and manual curation of networks	Curated network views
Chemical space organization	Qemistree (v1.0) [65]	Chemically informed trees linked to metadata	Chemical tree + metadata mapping
In silico annotation (formula/class/structure)	SIRIUS/CSI:FingerID (v5.8) CANOPUS (v1.0) [51]	Formula/structure/class prediction beyond library matches	Putative IDs/classes (confidence-labeled)
Spectral analogue search	Spec2Vec (v0.6) [66]; MS2Query (v1.1) [67]	Retrieve structural analogs beyond exact library matches	Analog candidates + similarity scores
Functional prioritization	Docking workflows (e.g., AutoDock Vina (v1.2.3))	Rank candidates for experimental validation	Ranked compound list

## Data Availability

Dataset is available on request from the authors.
